# Changes in Hungarian academic psychology after the end of “people's democracy”

**DOI:** 10.1002/jhbs.22231

**Published:** 2022-10-01

**Authors:** Csaba Pléh

**Affiliations:** ^1^ Department of Cognitive Science Central European University Budapest Hungary

**Keywords:** Bologna system, cognitive science, grant systems, Hungarian psychology, liberalized curricula, Westernization of Hungarian psychology

## Abstract

The paper surveys the last 30 years of Hungarian academic psychology. Around 1989–1990, the time of the great social changes Hungarian psychology was rather Westernized, but still a relatively small scientific field and applied profession. The opening and liberalization of politics made psychology in Hungary a booming profession and a rich research field. Education of psychologists was spreading, and becoming more Westernized in textbook usage and reading materials. Entrance numbers at two universities with 80 students were replaced by 2010 by 6 university programs and about 8000 incoming students. The training system is a Bologna type BA + MA + PhD system, The educational booming has its own problems. As all university subjects, psychology training is also underfinanced, with high teaching loads and a move by university management towards applied areas, neglecting basic research. The research activity is characterized by a fivefold increase of English language publications coming from Hungary over a 20 years period. University research was strengthened, and competitive grant systems were introduced, whth good success aretes by psychologists. Here again, managerial thinking questions many aspects of basic research and liberalized science management. These factors are peculiar to psychology, but they do have an impact on it. The paper gives some details about one chapter of academic psychology, cognitive psychology. Institutionally, support by the Soros foundation in the 90s for the university cognitive programs had as one consequence that three departments of cognition are active in Budapest today. Another aspect of insitutional development was the series of multidisciplinary conferences in Hungary (MAKOG), and Hungarian involvement in international graduate training programs in cognitive science. The most successful cognitive group, at Central European University (5 ERC grants, publications in leading journals) is recently chased out of Hungary by anti‐Western and antiliberal legal moves. This would certainly have a detrimental effect on Hungarian cognitive psychology for quite a time.

## 1 THE BACKGROUND. HUNGARIAN PSYCHOLOGY AT THE END OF THE 1980s

Hungarian psychology in the mid‐1980s was more Westernized, less ideologically dominated or infiltrated compared to most other Soviet dominated East European countries, and it was less ideologically penetrated than philosophy and some of the other social sciences like economy, history, or literary studies. This gives an internal context for the changes after 1989: while there were many changes, they have been less dramatic and in several regards they were continuations of earlier trends. Psychology was “oppressed” as a questionable science and ignored in social practice mainly in the 1950s (C. Pléh, 1997, 2017; Szokolszky, 2016). From 1963 on, a relative political neutralization was initiated in initiated in Hungary (Romsics, 1999), giving amnesties, for example, to most of the political prisoners sentenced for their participation or support of the 1956 revolution, developing trade and cultural relations with the US and most of West European countries (but for a while not with West Germany). At the same time the leading role of the communist party remained there in cultural life as well with a socialist‐communist version of the repressive tolerance described by Herbert Marcuse (1964) for industrialized Capitalist societies. Psychology has become a tolerated, but not a preferred domain in faculties of humanities in the 1960–1970s. As a small part of this “opening” process the *Hungarian Psychological Association* was reestablished, its journal also restarted in 1960, and in 1963 professional education with a licensing degree program in psychology started with applied training in mind (Laine‐Frigren, 2015). The 5 year training was divided into two parts. The first 2 years concentrated on biological fojn dations, statitistics, and experimental psychology. This was followed durng the next 3 years by personality, developmental and social psychology, and a specialization into one of three applied domains: clinical, industrial, and educational psychology.

C. Pléh (2017, p. 183) even tried to show stages in this process of reestablishment of psychology in Hungary during this time. The proposed stages are: reemergence (1960–1970), spread and differentiation (1970–1980), and social acceptance and less ideology (1980–1990). Westernization during these three decades was shown for example, among other things, by the change in references. In the main Hungarian language psychology journal in the international references from the late 1960s on, and in book reviews from the mid‐1970s, the initial dominance of Russian sources was declining, and English literature as a frame of reference dramatically increased (C. Pléh, 2017, pp. 190–191). This entire process of Westernization is not interpreted as a positive by all. Kovai (2017) for example treats it as a “self‐colonialization” process with negative connotations of mimicking the West, and ignoring local social problems in Hungarian psychology. In my view, Westernization corresponded to an individualizing modernization in social life. You may not like this, but this is certainly the process that has given place to the birth of modern psychology all around the Western world.

This Westernization was constrained and only relative in a repressive tolerant manner. Nonparty members, for example, could gain professional leadership positions, but under strict HR control from different levels of party leadership. International relations were allowed but again under strict control. (See about this as a eneral issue the analysis of Szakádát & Kelemen, 1992 regarding the Hungarian communist party based “nomenclature” system). All foreign publication manuscripts had to have a secret police controlled *nihil obstat* until 1989, but this control has become mostly formal. No one was reading your manuscripts anymore, the materials were just filed. Most psychologist have been government employees even if they worked in guidance services or in industry.

As for the content of academic psychology, one could summarize the situation by saying that you were not supposed to preach Marxist psychology, but you were not really allowed to speak against Marxism or the Party line in social issues either. Most of academic and professional psychology tried to neutralize psychology from the ideological context, approaching psychology to natural sciences and medicine. In this process of neutralization, there were still ideological infights between mainly Frankfurt school and Vygotsky inspired efforts to create a Marxist psychology and the mainstream professional trends (see Erős, 1991 for an original effort, and some of the papers in the volume edited by Borgos et al., 2019 for more contemporary continuations). Even in the modernized and professionalized context of socialist Hungary, psychoanalysis had a difficult time. Psychoanalytic practice was only gradually becoming officially tolerated. And for example the single most symptomatic communist party intervention in the 1960s into university curricula at Loránd Eötvös University in Budapest was related to psychoanalysis appearing as part of a course on personality, where the teacher (Magda Marton) had to drop the course and leave the faculty. No Freud book appeared in Hungarian until the mid‐1980s. The histrtoian of Hungarian psychoanalysis, Judit Mészáros, (2010, 2012) gives a detailed account of this. At the same time, both in child guidance and mental health care state run and financed support systems integrated most of applied psychology, with much of “dyamaic psychology” content included.

## 2 SOURCES USED IN THIS ACCOUNT

For the changes starting with 1989–1990 there are fortunately several sources available in Hungarian. The *Hungarian Psychological Association* had two thematic sessions in the mid‐2010s as part of their annual conference, discussing the history of Hungarian psychology mainly during the last half century. This was published as a bulky Hungarian volume edited by Cs. Pléh et al. (2019). Second, as part of a general overhaul of the last 30 years of Hungarian science, the *Hungarian Academy of Sciences* composed an internet resource in Hungarian (Falus, 2022). As part of this project, psychologists surveyed their own field in 15 chapters. That is also available in Hungarian as an internet resource (Urbán & Molnárné Kovács, 2022). The most important parts of the psychology survey are also published in the *Hungarian Journal of Psychology*, also in Hungarian but with extensive English abstracts. Most of the conspectus here relies on the substantial material in these surveys.

## 3 THE SOCIAL IMAGE OF PSYCHOLOGY CHANGING

In the changes appearing after 1989 general social, political and economic factors and psychology specific ones could be differentiated. Rainer (2017) is a god source about the interpretation of the structure and moving causes of these general changes. I use the accepted interpretation of the changes as a *liberalization*, mainly regarding changes that had a direct effect on science and intellectual professions. For two decades, between 1990 and 2010 this meant a professionalized depolitization of science and professional practice in psychology as well, as a continuation of depolitization in the 1980s.

1. *Political liberalization*. Multiparty system, with no more political commissars or even political parties at work places.
2. *Westernization* (nato, eu), including integration into international science exchange and financing systems. People and books freely travel.
3. *Changes in resource allocation*, increase of the inportance of research grant systems.
4. *Liberalization of higher education*. University autonomies. New disciplinary programs, new universities. This had an impact on philosophy, and many social sciences, business, sociology and social work, political science, communication prograns started to flourish.

Psychology in Hungary after 1990 on the levels of university education, research organization, public opinion, and even high politics has turned from being a tolerated poor relative into a shining new star of the human and social sciences. Psychology at the universities was mainly treated as a minor department by powerful historians and literary folks in humanities faculties of the 1970s. The increasingly more and more popular psychology from the mind 1990s started to support financially the humanities faculties. This is related to the fact that in the midst of much debated micromanaging and political discussions and tensions over the exact forms, Hungarian governments (both conservative and socialist) introduced into higher education from the early 1990s on a normative financing system. Universities have their most important budgetary allotment according to the number of their students with differences among the fields of the *per capita* amount (V. Berde & Vnyols, 2008). The government steps in by quaotas for different fields, but still the interest of would be students towards the fields is a crucial factor. As a consequence, within most universities individual departments on their turn are financed according to how many credits they teach to how many students. Psychology as major has become very popular, bringing in much credit moneys, plus in many large universities, psychology has an extra income through its involvement in teacher training. Thus, rather than psychology begging for money, from the mid‐1990s on, other departments are begging for financial solidarity from the psychologists in many humanities faculties.

This emancipation of psychology was not only financial, but also went on in *the symbolic aspects of science*. Psychology was a mainly tolerated domain in the 1970s in the eyes of party leadership and science management. Take the example of the Hungarian academy, that still is a central field of scientific reputation in Hungary. There was no psychologist member of the Hungarian academy of sciences between 1949 and 1985, when the social psychologist Ferenc Pataki was elected. Now, in 2022 there are five psychologist members of the Hungarian Academy of Sciences and psychology has a rather good representation compared to other social sciences. Pataki himself has been section chair and vice president of the Academy, another social psychologist, György Hunyady acting as section chair, and some others also filling leading national higher educational functions as well, such as Valéria Csépe chairing the National Gigher Education Committee (MAB under WEB pages).

Psychologists have become visible even as high level government leaders. On the conservative side, Pál Rókusfalvy the industrial and sport psychologist was under‐secretary for youth in 1990–1991 in the first postcommunist national conservative government in the Ministry for Public Welfare. On the left‐liberal side, George Csepeli, a professor of social psychology was political state secretary between 2002 and 2006 in two socialist‐liberal governments in the Ministry for Informatics and Telecommunications.

## 4 CHANGES IN THE EDUCATION OF PSYCHOLOGISTS

Let us consider first the quantitative aspects! Several universities started to initiate many new programs based on the perceived social popularity or perceived one sidedness (too theoretical, too Marxist) or feeling excluded from the programs in Communist times. New (non‐Marxist) programs of philosophy, sociology, anthropology, communication emerged (to be later curtailed by the managerial leadership of the ministry of education), and new universities were launched based on regional and religious interests. In 1989, at the end a 25 years socialist development, two degree programs of psychology with 5 year training were offered, for a few dozen students, one in Budapest (Eötvös U.) and one in Debrecen (Kossuth U.) After 1989, among the newly spreading programs psychology was the most popular one, and its public popularity and promotion by university management remained constant through three decades. The intention of university management and the public interest meat with the dissatisfaction of psychologists (both academic and professional) who considered themselves to be excluded from faculty positions in the socialist times. This later feeling especially characterized colleagues working at the research Institute of Psychology of the Hungarian Academy of Sciences, who provided thence the core faculty at some of the newly emerging psychology programs, for example at Janus Pannonius University of Pécs.

In the process of spreading of psychology programs by 1992, beside *Eötvös Loránd University*, Budapest and *Kossuth Lajos University*, Debrecen, *Janus Pannonius University of Pécs* also started a psychology degree program. By 1995 the intake of new students doubled compared to 1990, from 80 to almost 200. By 2000, six psychology degree giving universities showed up in the Hungarian higher education palette and they continue to function in 2022. *Attila József University* of Szeged joined the club, first in 1996 as a subsidiary of Debrecen, and from 1999 on its own right. Two denominational universities also started to give psychology degrees. In Budapest, *Pázmány Péter Catholic University* and *Károli Gáspár University of the Reformed Church* (Calvinist).

The student numbers were constantly increasing. By 2010, incoming students were competing for 800 slots. Until the mid‐1990s these are numbers under general ministerial control, from then on they are mostly based on decisions by autonomous universities. The 50 times increase in 35 years is remarkable. The average yearly increase was 12% (C. Pléh, 2019, p. 156). These numbers refer to state financed places. During the last decade the numbers increased by a further self‐financed quota of students, and at several training places English language programs for international students were also started at Eötvös, Károli, and Pécs universities.

Psychology is peculiarly popular during the general increase of higher education. It should still be seen in the broader context. After the 1990s, connected to the changes in the political system, there was an enthusiastic increase based on the political opening in Hungarian higher education student numbers in general for 2 decades. According to the data of V. Berde and Vnyols (2008), between 1990 and 2005 the entire Hungarian higher education influx showed a 3.5 increase. Psychology should be seen in this context. During the same time psychology entrance numbers showed a 7.5 increase. Thus, the popularity of psychology is very high compared to other fields. In recent application numbers (2020) psychology is the second most popular subject after business.

At the same time, this remarkable increase did not go without problems. That is true for the entire higher education system. As data from the National office of Education analyzed by E. Berde (2003); V. Berde and Ványolós (2008) showed as a crucial factor or consequence of the underfinancing of higher education after 1990 the faculties proved to be insufficient in the entire system. The underfinancing is sometimes accompanied by fights over tuition. In principle, Hungarian conservatives want no tuition, while liberal‐left wing politicians want tuition. In reality, however, witgh the increased of “self‐financed” students, about 25% of students pay tuition.

A crucial aspect of underfinancing is the teacher–student ratio. In 1990, the teacher to student ratio was 8, and by 2005 it was 16. V. Berde and Ványolós (2008) showed that this is a trend characterizing all Eastern Europe, with increasing class sizes and recently in Hungary sometimes 12–14 weekly teaching hours for faculty. The Law of Higher education lists 8 h for full professors, and 12 h for assistants. We do not have the actual numbers broken down for psychology, but it certainly is of the same magnitude. A firther factor is that psychology programs usually are parts of the humanities faculty in Hungary. In this context, the general underfinancing and insufficient faculty is also more specifically combined by a shortage of classroom and lab places, and shortage of extramural internship places. The underfinancing, sometimes accompanied by political fights over the issue of tuition, is a general characteristic of higher education in Hungary, it remains true both under left/liberal and conservative nationalist governments.

Thus, in particular for our profession and science, we should not have a rosy picture of the increasing educational interest towards psychology in Hungary. It was certainly a difficult task to create four full fledged new teaching faculties over a single decade, between 1996 and 2005. The faculty coming from the academy research institute certainly helped. They provided core faculty of experimental psychology in Debrecen and Pécs, and in social and developmental psychology at Pécs. There was also a shortage of leaders. Just to indicate with 1 number, in early 2022 out of the 6 psychology training university institutes with their roughly 30 departments, 3 institutes are chaired by teachers who are not full professors. That is rather strange in light of Central European traditions. At the same time, while three of these institutions have English language programs, and all of them do participate in Erasmus and other cross European exchange programs, mainly exchanging students, there is practically no non‐Hungarian faculty in any of them. Thus, internationalization is mainly true on the student andf research levelés, but not on the level of faculty. At the same time, while in the 1990s basic academic foundations were strong assets of the then existing programs (and were duly criticized for that by the practicing psychologists), by today four of the six programs (Debrecen, Szeged, Pázmány, and Károli) do not have any serious experimental psychology teaching faculty.

## 5 THE CONTENT OF PSYCHOLOGY TRAINING

The modern psychology program initiated in 1963 in Hungary aimed to include professional training as part of the 5 year curriculum. This was soon changed, and from the mind 1970 s there was a separation of basic level diploma and specialist training.

Many new developments after 1989 concerned the content and organization of the 5 year basic level training. On a general level, curricula have been liberalized as to their content, but at the same time, with a national quality assurance systems gradually put in place, the organization has become gradually less open and most importantly, less flexible compared to the early 1990s. The early 1990s offered much choice among special topics, and even choice of instructors at some places. That was the happy 5 years of sudden liberalization.

There were many changes in the accession of books and journals, Gradually, compared to the 1980s, where every individual book was a treasure, Hungarian psychologists were more and more able to read all relevant literature. One sign of the basic liberalization of content is *the use of textbooks*. In most broad areas, American textbooks are still used in translation. This is true for introduction, where the Hilgard‐Atkinson book is still in the market (I am using always the version that was translated to Hungarian, in this case Nolen‐Hoeksema et al. 2003). Incidentally, it is the most popular Hungarian language psychology book, with sales over 80,000 copies. There is a series of successful general textbooks. In developmental psychology, the Cole and Cole (1996) book, in social psychology Smith et al. (2015), in personality Carver and Scheier (2000), in clinical psychology Comer (1998). There are of course new textbooks written by Hungarian authors as well. I am only listing this sample of introductory American sources to show the extent of Westernization of content.

Hungary as the single East European country took part in the formation of the joint framework for psychology training in Europe (EuroPsyc, Lunt et al. 2005). That helped consciousness raising about the curricular organization of university programs in psychology inside Hungary as well. Hungarian pscyhologist can obtain the EuroPsy certificate through the Hungarian Psycholgical Association. Most importantly, the EuroPsy participation helped to keep the “psychologist” label for colleagues with a 5‐year training.

Another important factor was local, and related to regained university autonomy. On the initiative of György Hunyady, a leading social psychologist and in the early 1990s dean of humanities faculty at Eötvös University, as part of a general liberal revision of training at humanity faculties, a credit based flexible system with theoretical and applied specialization from Grade 3 was introduced in psychology. Hunyady (2019) described himself this process and the later organizational development leading finally to the creation of a new faculty of Education and Psychology at Eötvös University. The system initiated by Hunyady was a *de facto* two level system, preceding the introduction of the Bologna system of BA and MA in psychology. It was, however a 2 + 3 year system (rather than the Bologna 3 + 2), and in its intention it contained much flexibility and freedom and responsibility allowed for the students, than the later, now existing Bologna system.

In the mid‐1990s, an originally university initiated and self organized national higher education program accreditation system took shape in Hungary. This had impacts on psychology as well, surveying all existing and new programs (see their homepage MAB, 2022). As part of the regained university autonomy, PhD programs were formed at the universities, including psychology programs. Around 2005 all of this was fit into a “Bologna” type: BA‐, MA‐ PhD‐level training. six universities are giving BAs in psychology (called “behavior analyst,” while seven institutions give MAs in psychology. Beside the full fledged programs already mentioned, Budapest U of Technology and Economics (bute) also started to train industrial and cognitive science MA. There are four PhD training places in psychology (Eötvös, Debrecen, Pécs, bute). Training requirements were reformulated several times during the last 20 years. Parallel to this process, in governmental decisions central student quotas were abolished, which led again to a twofold increase of students in psychology, and to an increase in fee paying students around 2010. At the four universities giving PhDs in between 1993 and 2019, 396 PhDs were defended, with the bulk of them (42%‐, 165 candidates) at Eötvös University. The gender distribution mirrors better than before the actual ratios in the profession, 71% of the new PhDs being female (Demetrovics, 2019).

In the MA training level, there are seven different specialties, with a jointly developed roughly equivalent content at the different training universities. The most popular one is developmental/clinical at five universities. Clinical/health psychology is offered at four places, interpersonal/intercultural track is offered also at four places. Three universities offer cognitive, three social/organizational, three industrial/organizational and three guidance/school specializations. The postgraduate training requirements for psychologist specialist continue to be in place. Thus, taking an applied MA track does not make you a specialist. It is rather a preparation to look for employment in this field as a nonspecialist psychologist.

“Independent” applied work was only allowed for psychologists having obtained a specialized training based license, usually following a 2–3 year training. Already in the 1980s the specialist training usually went in training programs while the candidates are working in the given field. This is still true today. There are many refinements both on the level of specialist training and legal health regulations that allow, most importantly psychotherapy practiced by expert psychologists after sufficient training. Buda et al. (2009) show he difficult articulation of psychotherapy ractice in Hungary, while Harmatta (2019) follows the development up till today, and Szakács (2019) gives the legal background as well.

Though my paper does not go into the details of applied psychology changes two remarks are relevant about them. First, there is a clear renaissance of psychoanalysis both on the intellectual scene and in widespread practice (Mészáros, 2010, 2012). Second, there is a continous downsizing of state run support systems. As a result, both in guidance, and clinical work and even in organizational psychology private practice has become dominant in the 2000s.

Together with the general liberalization of the content of teaching, *management* remained either amateurish, or in the hands of managers inherited from the previous times. Similar to other leaders at the same university, for example, the two initial deans between 2003 and 2015 of the newly founded Faculty of Education and Psychology at Eötvös U, two psychologists, have been former influential Communist party members, I do not want to ostracize them by mentioning this, merely to indicate that there is an inertia of university leadership. The implication was that only who showed how to manage at other times were eligible managers in the new times. This is true according to Polónyi and Kozma (2022) for the entire Hungarian higher education system.

There is a built in tension with the new more professional management ideals and style fro the 2000s that is specifically felt in psychology. Ambitious managers of a newer kind emphasize grant related activities and scientific publications as the essence of university work today. For many mid career people at universities, this may lead to downplaying teaching activities. There is a constant tension between the teaching and research efforts. The only sensible way out of this trap is to decrease teaching load and allow for internal sabbatical like systems. At the moment, individual deals with faculty managers are in place instead of generic solutions.

Together with the general increase in university autonomy after 1990, the recent decade shows warning signs toward reorganization. Hegedűs and Polónyi (2015) two Hungarian higher education sociologists analyzed different aspects of university autonomy in Hungary between 1994 and 2014. In state universities, ownership and budget, student numbers and fees are usually not subjects of autonomous decisions, while hiring is autonomous, and curricular content is partly autonomous.

During the last 2 years, under the conservative nationalist government of Viktor Orbán these issues took an interesting new turn, where the results still have to be seen. Most of the state universities were suddenly privatized, but in a peculiar manner (see Higher Education Law under WEB resources). The newly nominated governing boards of the “private universities” are contemporary or previous members of the conservative government, or government affiliated business representatives, and the private universities shall have their state budget but on a contractual basis. It seems to be a strange centralized Thatcherite system, that combines the eternal East European dreams of centralization with the competitiveness and efficiency rhetoric of the new managerial class. One shall have to see if this will effect faculty hiring and curricula. The events at the university of theatre and film are warning signs: tensions between the new management and the faculty resulted in strikes, secessions, and quitting. But we do not see the consequences of this in psychology yet.

## 6 CHANGES IN PSYCHOLOGICAL RESEARCH

Beside the changes in the scientific qualification system (i.e., the reconstructed role of the universities in PhD training), and the introduction of an international style research committe evaluation system, three major factors characterize the changes of the research system of psychology after the fall of Communism. (1) International integration of Hungarian psychology, together with increasing internationally visible research output. (2) Strengthening the role of universities in research. (3) Introduction of competitive grant system for research financing.

### 6.1 Internationalization of Hungarian psychology

The acquired new freedom around 1989–1990 also meant further opening towards the West, but also increased interest on the part of would be Western partners. This was shown early on in many factors. A first factor was international psychology conferences being organized in Hungary around the time of the system change. Here are some examples. Already in 1988 a European Conference on Developmental Psychology was held in Budapest, on the initiation of the The International Society for the Study of Behavioural Development. Since then, with the establishment of the Cognitive Development Center at Central European University, Budapest has become a world center of developmental psychology conferences. Several hundred active participants from all the continents took part in the 12th annual bcccd meeting (bcccd22) with Fiery Cushman of Harvard University and Rebecca Saxe of MIT as invited speakers.

Hungarian psychologists along with linguists were invited to organize in Budapest the International Conference on Child Language on the invitation from the *International Association for the Study of Child Language* (IASC) in August 1990, and the 2nd European Congress of Psychology of efpa in July 1991. These invitations of course resulted from previous professional contacts, formed during late socialism, but they allowed many young people of the new generation to form lasting contacts. To take another example, in 1988, 1997, 2009, and 2019 the European history of psychology conferences took place in Budapest (cheiron europe and later eshhs), mainly due to the good professional social contacts and organizational role of the late Ferenc Erős, Zsuzsanna Vajda, and Anna Borgos in the responsible European organizations.

International conferences taking place in Hungary certainly helped to put Hungarian psychological research on the international intellectual map. Another important component was provided by individual and institutionally organized invitations and involvement of Hungarian researchers, teachers, and students. Such schemes are the ceepus from 1994, later the eu based erasmus scheme and the likes. Preceding these, in the 1990s, several unilateral sometimes generous efforts were also received to foster integration of Hungarian researchers and universities into the European schemes. In the cognitive section I list some examples but these were true for many areas, developmental, social, organizational. This was accompanied by integrating Hungarian psychologists also into the esf and erc systems, both as applicants and as evaluators, overseeing and initiating scientific cooperation, and creating new competitive grant systems.

The *increase of international (practically English) publications* was a characteristic consequence of the opening. During the decade of 1980–1989 on the whole 373 Hungarian psychological publications appeared in international outlets, in the next decade 558, and between 2000 and 2009, 2060 according to data form the PsychLit database (C. Pléh, 2017). This fivefold increase certainly was a cosmopolitan move. This is also shown by the PhD requirements, where while almost all dissertations are still written in Hungarian in psychology, international publications are a must by now.

The internationally visible Hungarian presence in psychology shows a 14‐fold increase over 50 years, from 1970 to 2020. It is still small, of course, as Hungarian research output in psychology represents 0.02% of the international output today as indicated by Schubert and Vasas (2010) as well as by C. Pléh (2017). In some regards, like absolute numbers weighted by the number of researchers, Hungarian psychological publication activity is less intensive compared to some of the neighboring countries, like the Czech Republic, but in some subfields, such as cognitive neuroscience and cognitive psychology researchers publish in higher reputation outlets, with more citations compared to the neighbors (Schubert & Vasas, 2010).

### 6.2 The strengthening of university research

On a lip service level, expectations during socialist times socialist times explicitly spelled out that research belongs to a university vocation. However, research was still concentrated preferably in the research institutes of the Academy, and only sporadic high standard individual efforts were visible at the universities in the 1960–1970s. This was the case for psychology as well. Changes began to appear already in the 1970s as a result of the ambitions of university faculties, and the newly established research support branch of the ministry of education. Alongside with this, some last efforts of the Communist science management allocated specific resources to foster university research (see the effects of these in psychology C. Pléh, 2017). After 1990, several national and international efforts were specifically introduced to increase university research. In psychology, this mainly facilitated initiating international cooperations and improved digitized technologies, both in teaching and research laboratories, gradually allowing fast internet access as well.

### 6.3 Formation of a national competitive grant system

Hungary as all Eastern European countries between 1950 and the 1970s was entirely characterized by a centralized planning system even of scientific research that supported institutions rather than individuals or projects. The young generation in the 1970s was only dreaming of grant systems where their projects could be financed instead of the state financed plan based research of the “old guard.” This system in particular was favorizing the research institutes of the Hungarian Academy of Sciences over universities, among them the Institute of Psychology. Moves towards a merit and project based grant system have taken place as part of the general Westernization of the country from the mid 1970s on first in the form of thematic grant systems, such as the system of Public Education and Disturbances of Social Adaptation grants (C. Pléh, 2017). This entailed more options for university based research.

By 1989, the centralized research spending was gradually decreased and the role of open grants and thematic grants, later many times tied to EU accession or EU programs was basically equalized. A central role was played in this process by the OTKA national research grant system (see about its ESF evaluation in OTKA, 2014). Psychology had a good success rate in this scheme, but with shaky administrative positions. It was usually aligned with education, but some of its chapters aligned with neuroscience. During the last 10 years, the Orbán government's R and D and I (innovation) policy favors innovation and applied research in all domains, with a less certain position of curiosity driven basic research. Parallel to this, governmental resource allocation moves back towards centralization and towards special thematic calls (National Innovation Office, 2013). Thus, in a particular manner, in the middle of efficiency and competition managerial rhetoric, a recentralization takes place in research resource allocation similar to what happens in university governance (see NKFI WEBsite).

## 7 A CASE STUDY: HUNGARIAN RESEARCH IN COGNITIVE PSYCHOLOGY

As an example for some of the changes in actual research practice during this 30 years, I present the fate of Hungarian cognitive psychology that I am most familiar with in Hungary. Both *institutionally and in personal regards* the Hungarian cognitive movement started off from good positions in the late 1980s. As part of the the general Westernization of Hungarian psychology the would be or in the mid‐1980s already existing cognitive teams had especially many active international relations, obtained many scholarships and sabbaticals in Western Europe and North America. This already established open working style provided for continuous presence in international science, in congresses and publications. This was also true for the education of a new generation of cognitive researchers. Cognitive psychology starting at Eötvös University in the form of seminars that were in fact elective classes in the more flexible psychology curriculum started to attract quite a few graduate psychology students, but also philosophers, neuroscientists, ethologists and evolutionary biologists, and even engineers and computer scientists. Gradually, a real interdisciplinary community took shape. In the 1990s this process was facilitated by the different varieties of the Soros Foundation acting in Hungary. The different organizational and legal versions of these influential interventions consisted of a series of good will organizations, some of them directly targeting specifically social sciences in Hungary. This foundation network financed longer graduate or postdoctoral training abroad of Hungarian cognitive researchers as well such as Ilona Kovács, Zoltán Nádasdy (Rutgers) Tamás Demeter, Mihály Racsmány (Bristol). Soros Foundation also supported tutoring activities at the elective small social science institution *Invisible College* and the early cognitive doctoral and graduate programs launched at Eötvös University and at Attila József University at Szeged between 1996 and 2001. In the frame of these programs, a few dozen then very up to date mimeographed or xerox copied advanced English language readers in cognition were produced and widely used as teaching materials. The grants also allowed the invitation as guest teachers or invited lecturers many excellent international scholars and Hungarians working abroad from British, American, Austrian universities (Steven Harnad, Alan Baddeley, Luca Bonatti, Martin Prinzhorn are examples for the first, and Gergely Csibra, Ilona Kovács, József Fiser, Zoltán Nádasdy for the second category).

The multiplicity of international contacts also resulted many movements of migration, partly due to the attraction of foreign cognitive PhD programs and universities attracting the just established young scholars abroad. Unlike the Communist times, however, this time moving to the West did not mean to become alienated from the Hungarian scene. There was continuous cross talk, and in the 2000s repatriations have started. At Budapest University of Technology and Economics Ilona Kovács, Zoltán Jakab, and Anna Babarczy showed up from the US and UK on the invitation of Csaba Pléh. Later, in the 2010s doctors and postdoctors who graduated and worked abroad showed up in Hungarian universities, mainly at Eötvös University, as Kristóf Kovács, Balázs Aczél, Attila Keresztes, Zoltán Nádasdy, partly or entirely continuing their research carriers in Hungary.

A new American graduate private university, Central European University was operating in Budapest from 1993, founded by George Soros. This university established around 2010 a Department of Cognitive Science. This has become a center of excellence in the field. Among its international and multidisciplinary faculty quite a few were repatriated Hungarians. George Gergely who came back earlier moved to CEU, as well as the other founder of the Baby Lab at CEU, Gergely Csibra. Two Transylvanian Hungarians joined them who graduated in Trieste with Jacques Mehler, Ágnes Kovács M. and Ernő Téglás. József Fiser and Máté Lengyel coming to the CEU from the US and from UK developed a vision lab and a computational modeling track.

All of this resulted by Budapest having three departments of cognition around 2020. Cognitive Science (2005) at the Budapest University of Technology and Economics and at Central European University (2010), and a Department of Cognitive Psychology (2010) at Eötvös Loránd University.

Another institutional aspect going along with the liberal political atmosphere was the *creation of private foundations and associations fostering cognitive activities*. The Hungarian Cognitive Science Foundation established in 1993 on the initiative of György Kampis, Vilmos Csányi and Csaba Pléh (MAKOG, 2022) has played a central role in interdisciplinary cooperation by a series of yearly winter conferences, putting cognitive psychology as everywhere in the world into a wider crossdisciplinarity network. In the 1990s, for the new generation being socialized in cognition these conferences were central in creating personal and disciplinary contacts. In 2017 the 25th makog conference took place. This foundation was fostering along with Eötvös U from 2004 the *Budapest Semester in Cognitive Science* a semester abroad program mainly for American students offering cognitive science courses in Budapest, taught by Hungarian instructors (for more details see Pléh et al, 2017; Pléh and Racsmány, 2021).

Both established and newly formed publishing houses developed extensive psychological profiles, with dozens of new Hungarian textbooks, hundreds of translations, and many dozens of pop books. in this process, most of ambitious psychologists in the new area developed good and intense contacts with Hungarian publishers. This was true for the cognitive domain as well for publishing original research. Akadémiai Kiadó of Budapest even sponsored a book series in English entitled *Neurocognitive Development and Impairements*. Cs. Pléh et al. (2013, 2014) are two good examples for their conference based publications of two events that took place in Hungary.

Among the new publishers, Osiris Publisher brought out the best textbooks. In their psychological paperback translations from the cognitive field books by Changeux, Clark, Dehaene, Dennett, Donald, Sperber, and Tomasello appeared. Typotex Publisher launched a series edited by Ilona Kovács and Csaba Pléh and entitled *Body and Mind*. Beside Hungarian authors like Tamás Bereczkei and Csaba Pléh, books by Changeux, Dennett, Gould, Jouvet, Julesz, Pinker, and Karl Popper were translated in this series. Gondolat (Thought) Publishers carries a Cognitive Seminar series with Hungarian authors about 20 volumes by now, publishing most of the makog proceedings as well, and in a series of *Knowledge‐Theory‐Culture* books by Bruner, Canguilhem, Changeux, Cole, Mehler were translated.

Hungarian cognitive psychologists participated in international cognitive graduate training efforts, both as students and as instructors. Some of these have an interesting central European focus. Students and instructors participated in the cognitive science summer schools initiated by the late Boicho Kokinov at New Bulgarian University in Sofia. In 2022, they will held the 27th of these schools. Hungarian cognitive psychologists took part in the elaboration of the new Middle European Interdisciplinary Master program for Cognitive Science (MeICogSci), and together with Vienna, Ljulbljana, and Bratislava Universities, Eötvös University takes part in this program that offers joint courses and guest semesters for students (https://www.meicogsci.eu/).

Hungarian cognitive psychologists take part in the organization of many international conferences of their own field, showing their recognition. In 2004, the European Conference on Visual Perception took place in Budapest. As most notable among them was in 2013 the 18th congress of the European Society for Cognitive Psychology (escop) that took place in Budapest organized by BUTE and CEU, with abut 1000 participants. As a continuation of their already existing networking activities, in 2006 Melita Kovačević (Zagreb U, and Ilona Kovács and Csaba Pléh (bute) established the Central European Cognitive Science Foundation (cecog). This Budapest chartered institution has the main function of organizing the yearly ducog conferences every May in Dubrovnik with about 100 participants. Invited keynotes present a special topic, but the poster sessions welcome presentations from cognitive psychology and cognitive science at large. In 2022, the 13th ducog had the lead topic of Cognitive and Functional Perspectives on Emotions.

Hungarian cognitive researchers as part of their success, obtained many notable research grants from outside Hungary. As co‐PIs they took part in several NSF grants. During the last 5 years they obtained 5 ERC grants. The excellence of the CEU department is shown by the fact that all of these grants were obtained by them in a very competitive system, while no other Hungarian cognitive group was successful. This very sadly shows that the government enforced relocation of Central European University to Vienna shall have a very detrimental effect on level and organization of Hungarian cognitive research (see CEU under WEBsites).

## 8 SUMMARY AND OUTLOOK

In short, Hungarian psychology from 1990 showed a continuous Westernization and internationalization. This was true in university teaching, in the increasing sophistication and international reputation of research, and the social acceptance of psychology as a leading social science. During the last 10 years, there are some tension in this continuous development. The underfinancing of the university system, the increasing teaching load, and the managerial concentration on applied research are not specific to Hungary and to psychology. Most of the recent Hungarian tensions are similar to events happening over university management and the fate of curiosity driven research in most of the Western world. The particular Hungarian feature is, however, the dislike and even persecution of liberal ideas and institutions by the Orbán goverment. That is creating specific threats to psychology a science and profession that is mostly aligned with liberal social philosophy and certainly with the primacy of individualism on the ideological level.

## Data Availability

The data that support the findings of this study are available from the corresponding author upon reasonable request.
